# Birthweight correlates with later metabolic abnormalities in Chinese patients with maturity-onset diabetes of the young type 2

**DOI:** 10.1007/s12020-019-01929-6

**Published:** 2019-04-26

**Authors:** Junling Fu, Tong Wang, Jieying Liu, Xiaojing Wang, Ming Li, Xinhua Xiao

**Affiliations:** Department of Endocrinology, NHC Key Laboratory of Endocrinology, Peking Union Medical College Hospital, Chinese Academy of Medical Sciences and Peking Union Medical College, Beijing, 100730 China

**Keywords:** *GCK •* MODY2 *•* Birthweight *•* Metabolic abnormalities

## Abstract

**Purpose:**

Glucokinase-maturity onset diabetes of the young (*GCK*-MODY), also known as MODY2, is caused by heterozygous inactivating mutations in the *GCK* gene. The aim of this study is to investigate the relationship of birthweight and cardiometabolic characteristics in MODY2 patients.

**Methods:**

Genetic screening for *GCK* mutations from 192 classical MODY families was performed, and birthweight and clinical profiles of 76 patients from 25 families with identified *GCK* mutations were collected.

**Results:**

Mutations in *GCK* were identified in 25 (13%) of the 192 families. Four novel (c.1334 G > C, c.1289_1294delTGACGC, c.584 T > C, and c.30delC) and twenty-one previously reported mutations were identified and cosegregated with the clinical phenotypes of MODY2 within the pedigrees. MODY2 patients presented a mean birthweight of 3.11 ± 0.44 kg. Additionally, birthweight was negatively correlated with 2 h-postprandial glucose (*r* = −0.426, *P* = 0.006), glycated albumin (*r* = −0.462, *P* = 0.035), glycated hemoglobin (*r* = −0.529, *P* = 0.001), total cholesterol (*r* = −0.430, *P* = 0.016), and low-density lipoprotein cholesterol (LDL-C) (*r* = −0.383, *P* = 0.033) levels after adjustment for age, gender and BMI. Importantly, among the patients who inherited mutations from their mothers, 7 patients whose mothers were treated with insulin during pregnancy had particularly lower birthweight (2.83 ± 0.39 vs. 3.37 ± 0.39 kg; *P* = 0.003), higher total cholesterol (6.15 ± 0.43 vs. 4.06 ± 0.16 mmol/L; *P* = 0.002) and LDL-C (4.05 ± 0.35 vs. 2.21 ± 0.13 mmol/L; *P* = 0.001) levels compared to the other 21 patients whose mothers received no treatment.

**Conclusions:**

The correlations between birthweight and cardiometabolic indexes indicated that MODY2 patients with lower birthweight (<3.1 kg) should be monitored and treated more actively to prevent metabolic abnormalities, particularly dyslipidemia. Importantly, prenatal genic diagnosis is highly recommended to avoid inappropriate treatment in pregnancy leading to lower birthweight of offspring.

## Introduction

Maturity-onset diabetes of the young type 2 (MODY2) constitutes 10–60% of MODY cases, and is inherited as an autosomal dominant disease. This condition is characterized by mild fasting hyperglycemia from birth, which is diagnosed at a younger age than type 2 diabetes, and rarely develops chronic complications [1]. The disease is caused by a loss of function of the glucokinase (*GCK*) gene. GCK, a glycolytic enzyme, catalyzes the conversion of glucose to glucose-6-phosphate [2]. *GCK* is mainly expressed in pancreatic beta cells and hepatocytes. In beta cells, GCK serves as a glucose sensor, which plays a role in glucose-stimulated insulin secretion to mediate glucose utilization [3]. In hepatocytes, GCK is important for glucose uptake and glycogen conversion [4]. The prevalence of mutations in *GCK* is 1.1/1,000 in the white European population, and is higher particularly in women with gestational diabetes mellitus (2%), the higher prevalence during pregnancy may be due to screening for gestational diabetes, since most of the patients with MODY2 are still not diagnosed [5]. With the advancement of genetic testing technology and the in-depth understanding of MODY2, more than 600 mutations in *GCK* have been reported (http://www.hgmd.cf.ac.uk/ac/index.php), while increasing number of mutations in *GCK* have been detected in Asian MODY2 patients [6–8].

*GCK*-MODY is considered to be a stable state of hyperglycemia except during pregnancy. However, it has been suggested that the risk of insulin resistance is increased in individuals with MODY2 in the long term [8]. To our knowledge, there is no potential early marker to predict the progression of the disease.

Maternal hyperglycemia can stimulate the fetal insulin secretion, thus, leading to macrosomia. In the case of *GCK-*MODY patients, their birthweight hinges on whether maternal gestational diabetes was well treated. In other words, the birthweight of MODY2 patients largely depends on maternal glucose control. Babies with a *GCK* gene mutation who are born to a *GCK*-MODY mother with intensive glucose controlling during pregnancy will typically have low birthweights, consistent with the results of babies who inherit the mutation from their fathers [9]. Lower birthweight is considered as an independent risk factor for cardiometabolic risk profiles, including insulin resistance, diabetes and dyslipidemia [10–13]. However, whether it also plays a detrimental role in the development of cardiovascular diseases in MODY2 patients remains unknown.

In the present study, we conducted genetic screenings of possible Chinese MODY families to uncover additional novel pathogenic mutations in *GCK*. Furthermore, to clarify the effects of birthweight on metabolic profiles in MODY2 patients, we evaluated the association of birthweight with metabolic characteristics in individuals with MODY2.

## Materials and methods

A total of 192 unrelated pediatric, adolescent or young adult patients with clinical features of MODY were included in the study [14]. The inclusion criteria were as follows: (1) the age at onset of diabetes ≤ 45 years; (2) family history of diabetes in at least two generations with autosomal dominant mode of inheritance; and (3) absence of autoantibodies including glutamic acid decarboxylase antibody (GAD), protein tyrosine phosphatase antibody (IA2) and islet cell antibody (ICA); (4) nonobesity (BMI < 28 kg/m^2^). They were recruited from the out-patient clinic of Beijing Peking Union Medical College Hospital from January 2014 to December 2017. The families were all unrelated and of Chinese descent. Written consent was obtained from all participants or from responsible family members.

For mutation screenings, genomic DNA of the 192 MODY pedigrees as well as the relatives available from the 192 families (*n* = 395) were isolated from peripheral blood lymphocytes and used to screen *GCK* mutations by polymerase chain reaction (PCR) direct sequencing. Ten exons of the human *GCK* expressed in pancreatic beta cells and hepatocytes were screened using primer sequences designed by Premier 5 software (Supplementary Table 1). The complete coding sequence of the *GCK* gene was screened in probands by direct sequencing. PCR products corresponding to abnormal electrophoretic patterns were directly sequenced to characterize nucleotide variants on an automated ABI 377 sequencer (Perkin-Elmer Corp., Foster City, CA). The NCBI BLAST database was then applied to identify variants by aligning with reference sequences NM_000162. Databases including the Exome Variant Sever (http://evs.gs.washington.edu/EVS), dbSNP database in NCBI (http://www.ncbi.nlm.nih.gov/snp/) and UCSC genome bank were used to exclude single nucleotide polymorphisms (SNPs). In addition, the identified variants should not have been found in 100 healthy controls. The presence of mutations in the relatives of identified MODY2 patients was investigated by direct sequencing of the affected exons. The functional effects of variants were predicted by PolyPhen2 (http://genetics.bwh.harvard.edu/pph2/), SIFT (http://sift.jcvi.org) and Mutation Taster (http://www.mutationtaster.org).

The clinical and biochemical characteristics of patients with identified *GCK* mutations were collected. Clinical indexes including height, weight, systolic and diastolic blood pressure (SBP and DBP), inheritance of the mutations, history of diabetes, the current treatment for diabetes, and maternal treatment during pregnancy were recorded for each participant. Body mass index (BMI) was calculated as weight (kg)/height (m)^2^.

Information regarding birthweight was based on self-reports according to the parent-held medical certificate of birth, which was available in 65 subjects. Among the samples (*n* = 76), 57% were born in hospitals, 28% at home with professional help and 8% at home without professional help.

Venous blood samples were collected after an overnight (≥10 h) fast. Fasting insulin and C-peptide, as well as 2 h- postprandial insulin and C-peptide levels were measured by chemiluminescent analysis. HbA1c was measured using dedicated high-performance liquid chromatography. GAD and IA2 were determined by ELISA (SIMENS ADVIA Centaur XP, Germany), and ICA were measured using indirect immunofluorescence. The concentrations of plasma glycated albumin, high sensitive C-reactive protein (hs-CRP), triglyceride, total cholesterol, low-density lipoprotein cholesterol (LDL-C), high-density lipoprotein cholesterol (HDL-C), aspartate aminotransferase, alanine aminotransferase, creatinine and uric acid were assayed using the automatic biochemical analyzer (AU5800; Beckman Coulter, USA). Insulin resistance was assessed by homeostasis model assessment of insulin resistance (HOMA-IR), HOMA-IR = fasting insulin (mU/L) × fasting glucose (mmol/L)/22.5.

Analyses were performed using the Statistical Package for Social Sciences 21.0. Data exhibiting skewed distributions were logarithmically transformed prior to analysis. The results are expressed as the mean and standard deviation (SD) for continuous variables and counts and percentages for categorical variables. Differences in clinical features between subjects with stratified birthweight were tested using Student’s t-test and general linear model after adjusting for age, gender and BMI, and expressed as the mean ± SD. Partial correlation coefficients were calculated to evaluate the association between birthweight and continuous metabolic parameters after adjusting for confounders. Multiple linear regression models were used for metabolic traits in relation to birthweight, and the β coefficient and 95% CIs were calculated after adjusting for age, gender and BMI. A *P* value < 0.05 (two-sided) was considered statistically significant.

## Results

**Fig. 1:**
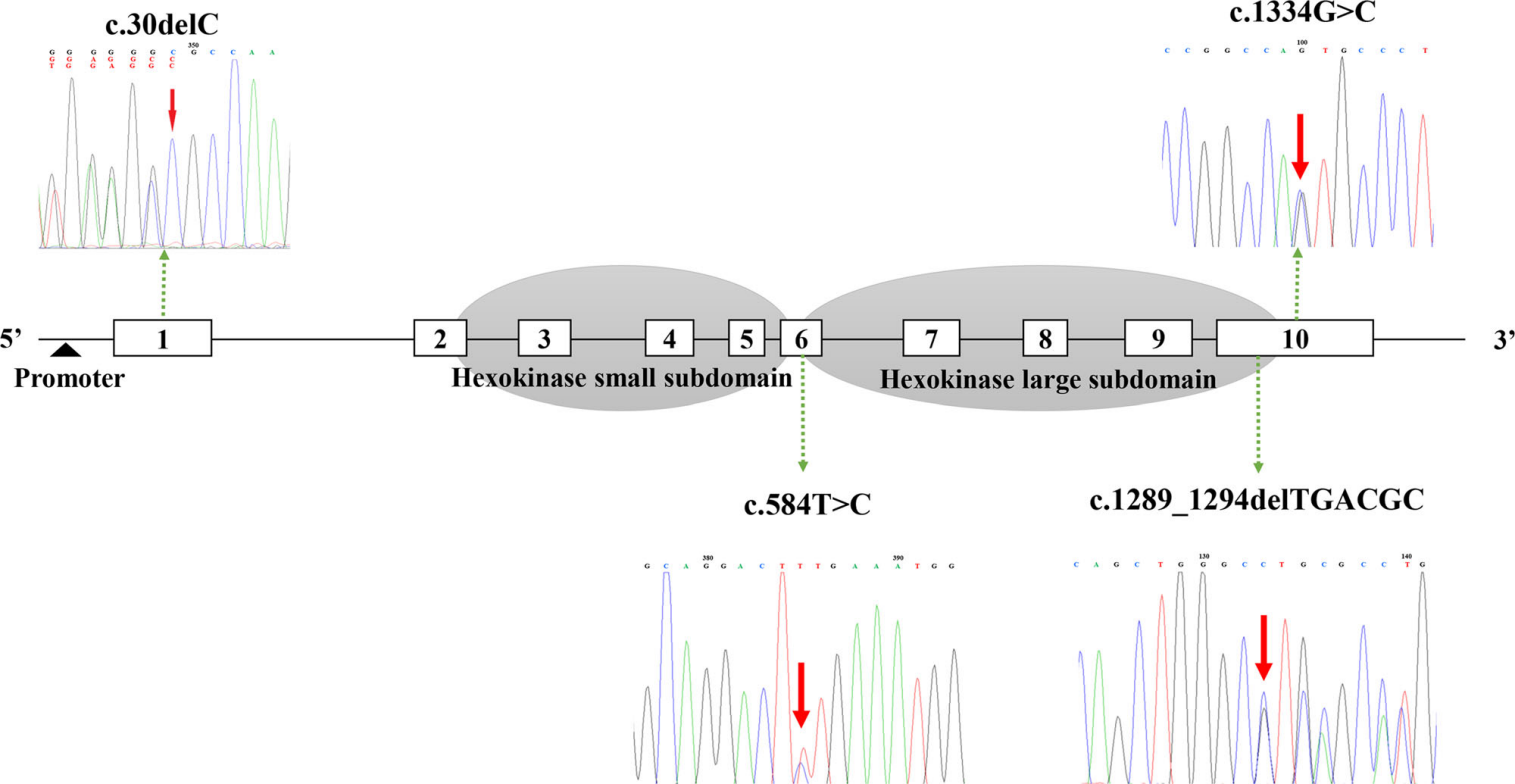
The sequencing chromatogram and position of the novel *GCK* mutations. Arrows indicate the changed nucleotide bases

In this study, a total of 25 mutations were identified in the *GCK* gene in 25 families (including 76 subjects) from a total of 192 pedigrees, representing 13% of our population. Four of the mutations were novel (c.1334 G > C, c.1289_1294delTGACGC, c.584 T > C, and c.30delC) and twenty-one were previously reported, and all of them cosegregated with the clinical phenotypes of MODY2 within the pedigrees. Detailed characteristics of these mutations are shown in Supplementary Table 2 and Fig. 1.

Carbohydrate metabolic traits were detected among the 76 *GCK* gene mutated cases with a mean age of 32.3 years old, and the mean duration of diabetes of the 76 patients was 4 years (Table 1). In general, 42% of the subjects were male, and the mean fasting and 2h-postprandial glucose levels were 6.84 ± 0.79 mmol/L and 9.59 ± 2.72 mmol/L, respectively; the mean fasting and 2-h postprandial C-peptide levels were 0.93 ± 0.40 ng/ml and 1.20 ± 0.55 ng/ml, respectively. Glycated albumin and hemoglobin A1C were moderately elevated (17.76% and 6.49%, respectively). Regarding the treatments, 91% of the patients were on diet control and exercise, whereas 4% were on insulin treatment, and 5% were receiving oral antidiabetic agents. A total of 37% of the mutations were inherited from mothers, while 24% were from fathers. Importantly, the mean birthweight of the patients was 3.11 ± 0.44 kg (range from 2.25 kg to 4.0 kg).

**Table 1:** Clinical characteristics of our MODY2 cases (*n* = 76)

Laboratory (serum)	Value (mean ± SD; *N*, %)
Age of MODY2 diagnosis (years)	32.27 ± 21.42
Sex (male/female)	32/44 (42%/58%)
Age of hyperglycemic onset (years)	27.12 ± 17.67
Duration of diabetes (years)	4.14 ± 5.01
Birthweight (kg)	3.11 ± 0.44
Birthweight Range (kg)	2.25–4.0
Body mass index (kg/m^2^)	20.05 ± 3.89
Systolic blood pressure (mmHg)	110 ± 18
Diastolic blood pressure (mmHg)	70 ± 12
Fasting glucose (mmol/L)	6.84 ± 0.79
2 h-postprandial glucose (mmol/L)	9.59 ± 2.72
Fasting insulin (mU/L)^#^	1.66 ± 0.73
2 h-postprandial insulin (mU/L)^#^	3.24 ± 0.84
Fasting C-peptide (ng/ml)	0.93 ± 0.40
2 h-postprandial C-peptide (ng/ml)	1.20 ± 0.55
Glycated albumin (%)	17.76 ± 3.08
Glycated hemoglobin (%)	6.49 ± 0.89
HOMA-IR	0.49 ± 0.20
Current therapy (Diet and exercise/OHA/Insulin)	69/3/4 (91%/5%/4%)
Inheritance (Maternal/Paternal/De novo/Unknown)	28/18/30 (37%/24%/0%/39%)
Total cholesterol (mmol/L)	4.22 ± 0.82
Triglyceride (mmol/L)	0.67 ± 0.39
HDL-C (mmol/L)	1.43 ± 0.27
LDL-C (mmol/L)	2.35 ± 0.75
Hs-CRP (mg/L)	0.38 ± 0.43
Alanine transaminase (U/L)^#^	2.68 ± 0.42
Aspartate aminotransferase (U/L)^#^	3.20 ± 0.49
Creatinine (umol/L)^#^	3.95 ± 0.40
Uric acid (umol/L)^#^	5.52 ± 0.28

^#^Skewed distributions were logarithmically transformed. Data were expressed as % or mean ± SD

*MODY2* maturity-onset diabetes of the young type 2, *HOMA-IR* homeostasis model assessment of insulin resistance, *LDL-C* low density lipoprotein cholesterol, *HDL-C* high-density lipoprotein cholesterol, *Hs-CRP* high sensitive C-reactive protein

The coefficients of partial correlations between birthweight and metabolic parameters are shown in Supplementary Table 3. Birthweight was significantly negatively correlated with 2 h-postprandial glucose (*r* = −0.361, *P* = 0.014), glycated albumin (*r* = −0.441, *P* = 0.019), glycated hemoglobin (*r* = −0.44, *P* = 0.003) and total cholesterol (*r* = −0.341, *P* = 0.039) levels. Furthermore, birthweight was marginally negatively correlated with BMI and LDL-C levels (all *P<* 0.1). After adjustment for age, gender and BMI, birthweight still showed a significant inverse correlation with LDL-C level (*r* = −0.383, *P* = 0.033), and the significant association with 2h-postprandial glucose, glycated albumin, glycated hemoglobin and total cholesterol levels persisted.

**Fig. 2:**
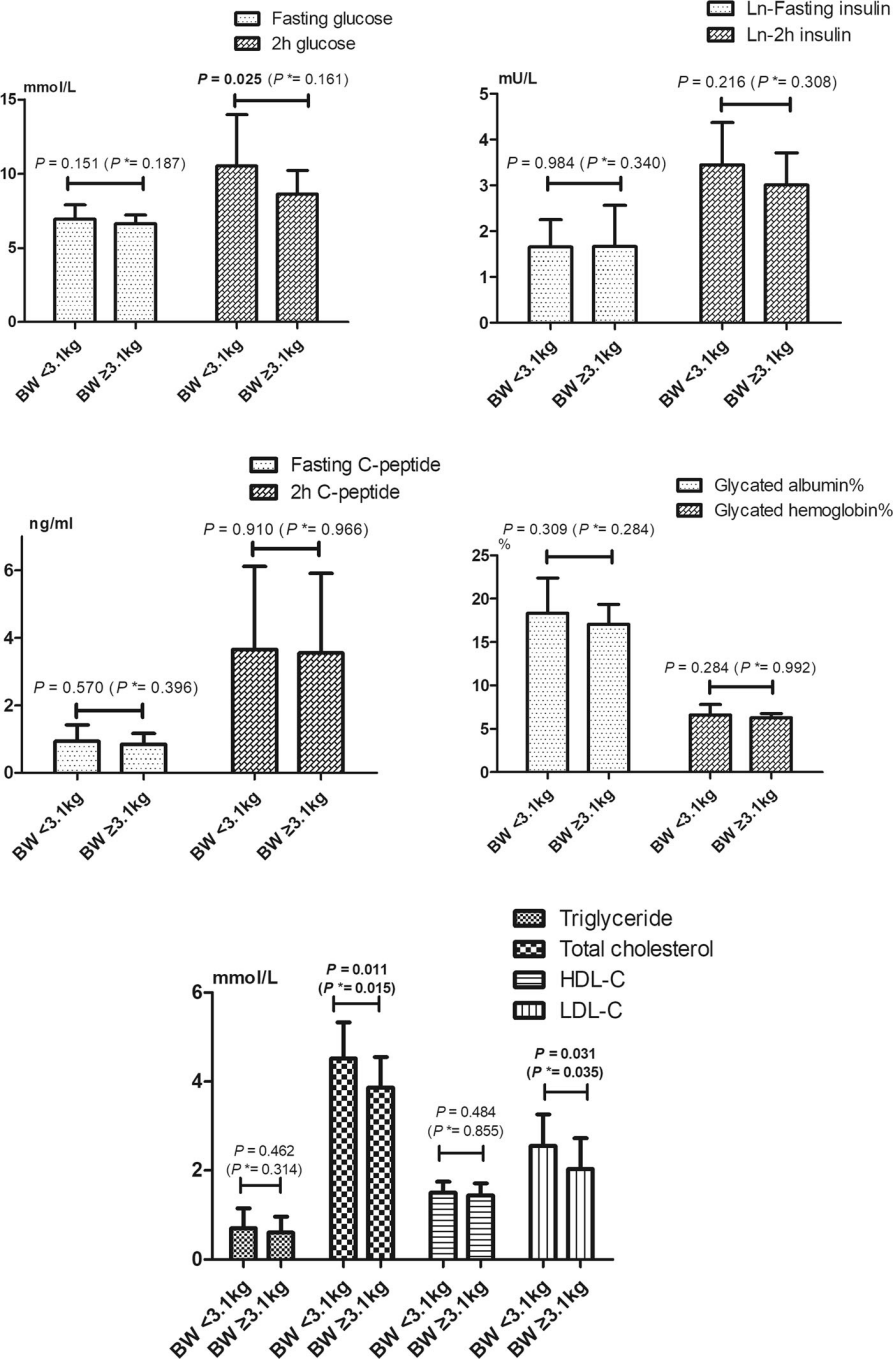
Comparison of metabolic traits across the two birthweight groups. Student’s *t*-test and general linear model were used in the analysis, and data were expressed as mean ± SD. *P was adjusted for age, gender and BMI. *BW* birthweight, *LDL-C* low density lipoprotein cholesterol, *HDL-C* high-density lipoprotein cholesterol

To evaluate the association between birthweight and metabolic traits, we classified the subjects into two groups based on the cut-off point of mean birthweight (3.1 kg), which are shown in Fig. 2. Participants with high birthweight (≥3.1 kg) showed lower concentrations of 2 h-postprandial glucose (*P* = 0.025), total cholesterol (*P* = 0.011) and LDL-C (*P* = 0.031). The lipids indexes (total cholesterol and LDL-C) in the two groups remained significantly different after adjustment for age, gender and BMI (all *P<* 0.05), while the difference of 2h-postprandial glucose was attenuated (*P>* 0.05).

In the analysis of multiple linear regression, shown in Table 2. Birthweight was negatively correlated with 2h-postprandial glucose (*P* = 0.006), glycated albumin (*P* = 0.035), glycated hemoglobin (*P* = 0.001), total cholesterol (*P* = 0.016), and LDL-C (*P=* 0.033) levels. Every 1 kg of additional birthweight was shown to yield 2.620 mmol/L lower 2h-postprandial glucose, 3.297% lower glycated albumin, 1.031% lower glycated hemoglobin, 0.769 mmol/L lower total cholesterol and 0.627 mmol/L lower LDL-C levels after adjustment for age, gender and BMI, the conclusion remained the same after further adjustment for the duration of diabetes.

**Table 2:** β (95% confidence interval) for metabolic traits according to birthweight levels after adjusted for age, gender and BMI

Variables	β (95%CI)	*P*
Fasting glucose (mmol/L)	−0.371 (−0.902–0.160)	0.167
2 h-postprandial glucose (mmol/L)	−2.620 (−4.450–0.791)	**0.006**
Fasting insulin (Mu/L)^#^	−0.008 (−0.723–0.707)	0.981
2h-postprandial insulin (Mu/L)^#^	−0.606 (−1.323–0.111)	0.092
Fasting C-peptide (ng/ml)	−0.047 (−0.225–0.130)	0.588
2 h-postprandial C-peptide (ng/ml)	0.405 (−1.184–1.994)	0.602
Glycated albumin%	−3.297 (−6.334–0.260)	**0.035**
Glycated hemoglobin%	−1.031 (−1.582–0.479)	**0.001**
HOMA-IR	0.009 (−0.186–0.204)	0.925
Total cholesterol (mmol/L)	−0.769 (−1.383–0.156)	**0.016**
Triglyceride (mmol/L)	−0.141 (−0.438–0.156)	0.339
HDL-C (mmol/L)	−0.029 (−0.262–0.205)	0.803
LDL-C (mmol/L)	−0.627 (−1.201–0.053)	**0.033**

^#^Skewed distributions were logarithmically transformed. Values in bold are significant at *P<* 0.05

*BMI* body mass index, *HOMA-IR* homeostasis model assessment of insulin resistance, *LDL-C* low density lipoprotein cholesterol, *HDL-C* high-density lipoprotein cholesterol

To further evaluate whether the source of the mutations had an impact on birthweight and clinical measurements, we performed a comparison of clinical parameters according to the origin of the mutations among 46 patients (Supplementary Table 4). Patients who inherited the mutation from their mothers had significantly earlier hyperglycemic onset (15.96 vs. 24.78 years, *P* = 0.027), younger MODY2 diagnosis age (18.21 vs. 28.81 years, *P* = 0.031), and lower triglyceride levels (0.55 ± 0.22 vs. 0.84 ± 0.47 mmol/L, *P* = 0.029) than others, while the difference in triglyceride levels disappeared after adjustment for age, gender and BMI (*P* = 0.141). No other difference was found between the two groups. Additionally, among the 28 patients who inherited mutations from their mothers, 7 patients whose mother were treated with insulin during pregnancy yielded particularly lower birthweights compared to other 21 individuals whose mothers received no treatment (2.83 ± 0.39 vs. 3.37 ± 0.39 kg; *P* = 0.003). In addition, these patients (*n* = 7) demonstrated significantly higher total cholesterol (6.15 ± 0.43 vs. 4.06 ± 0.16 mmol/L; *P* = 0.002) and LDL-C (4.05 ± 0.35 vs. 2.21 ± 0.13 mmol/L; *P* = 0.001) levels.

## Discussion

In this study of Chinese patients with clinical features of MODY, we identified 25 different *GCK* mutations in 25 families from 192 pedigrees (13%); four of them were novel mutations (2 missense mutations and 2 deletion mutations). All mutations found in our study were considered as pathogenic based on prediction analysis, and cosegregated with clinical phenotypes of MODY2 within the families. The most notable finding is that the birthweight of patients with MODY2 was negatively correlated with glucose and lipid indexes. Thus, our results showed that birthweight may be an early indicator to predict metabolic conditions in patients with MODY2. We found that optimal maternal glucose control was correlated with lower birthweight, which showed benefits to the neonates without mutations, and disadvantages to neonates with mutations, therefore, fetal genotype screening is recommended to avoid inappropriate treatment leading to lower birthweight.

### Mutations in GCK

In the present study, we identified four novel mutations and twenty-one known mutations were localized to exons 1–10. The *GCK* gene consists of 10 exons (NM_000162) and encodes a 465-amino acid protein with three tissue-specific isoforms due to alternative splicing at exon 1 [1]. There were twenty-four *GCK* mutations identified in our study localized to exons 2–10, which encode the functional structure domain of both the pancreatic and hepatic GCK isoforms. In addition to common mutations located in exons 2–10, we found a novel deletion mutation c.30delC occurring at the alternative splicing area in *GCK* (amino acid position 10 of exon 1) which was highly conserved across various species and absent in 100 healthy controls. Moreover, the mutation was regarded as a disease-causing mutation according to three bioinformatics tools. Up to now, there have only been 7 missense and 2 deletion mutations reported in exon 1 (grch37.ensembl.org/Homo_sapiens/Transcript/Exons). The patients carrying this mutation showed well-controlled glucose, and normal pancreatic islet function as well as normal lipid profiles without any treatments. Therefore, the results indicated that MODY2 patients with mutations located in both the functional structure domain and splicing domain demonstrated similar phenotypes. Overall, this study enriches our knowledge of the *GCK* mutation spectrum.

### Prevalence of MODY2

Herein, we found the prevalence of MODY2 in our study (13%) was lower than that in France (56%) [15], Spain (25–41%) [16, 17] and Italy (41–61%) [18, 19], but was similar to United Kingdom MODY2 pedigrees (12.5%) [20]. With respect to the Chinese population, the prevalence of MODY2 found in our study was comparable to the results of Limei Liu et al. (10.4%) [8], but was much higher than the rate in Hong Kong (1%) which used a relatively loose MODY criteria [21]. The discrepant results among and within countries might reflect the differences between races or criteria for MODY used in these research studies.

### Clinical characteristics of MODY2

In the present study, we described clinical characteristics in 76 Chinese MODY2 patients. Some of the results were similar to those previously reported, including the mild elevated fasting blood glucose, and normal lipid profiles, while a significant proportion of them (50%) did not meet the diagnostic criteria for diabetes [9, 22]. As reported by several studies, 2h-glucose in these patients yields small increases, which might be explained by the fact that *GCK* mutations do not reduce maximal insulin secretion [23]. For the treatment, in line with previous observations, most of our subjects (62.2%) maintained euglycemia without any medical therapy [9, 22]. Furthermore, the accurate diagnosis of MODY2 took an average of 4 years in our study, which is significantly shorter than the 12 years reported by a survey from the United Kingdom [24, 25], indicating the development of genetic diagnostic technology and in-depth understanding of the disease. The current study provides clinical information to identify patients with MODY2 in the Chinese population.

### Birthweight of MODY2

With respect to the birthweight of MODY2 patients, subjects in our study presented a mean birthweight of 3.1 kg which was in accordance with previously reported findings [9, 26]. Moreover, we confirmed a significantly higher birthweight in subjects who inherited the mutations from untreated mothers compared to those who inherited mutations from their fathers or treated mothers [9].

### Associations of birthweight and later metabolic characteristics in MODY2

Our previous study and others have suggested a robust association between low birthweight and increased risk of cardiovascular diseases [10–13]. The potential mechanisms may be based on the intrauterine reprogramming or the presence of genetic defects [27]. In the present study, even if the birthweight of the individuals included in our sample did not meet the diagnostic criteria for low birthweight (<2.5 kg), we found lower (<3.1 kg) birthweight was independently correlated with patients’ 2h-postprandial glucose, glycated albumin and glycated hemoglobin levels. In contrast, Velho G et al. [28] did not demonstrate a significant difference of metabolic phenotypes later in life in MODY2 patients with different birthweight. These disparate findings may be due to different analytical methods and ethnic differences. Since relevant data are still scarce [17, 29, 30], further large sample, multiracial, and prospective studies are needed to confirm the relationships of birthweight with later life metabolic phenotypes in MODY2 patients. Additionally, although it is well known that patients with MODY2 yield cardio-protective lipid profiles compared to T1DM and T2DM patients [31, 32], there were still 4% of *GCK*-MODY patients reporting with macrovascular disease in an adult *GCK*-MODY cohort [9]. Herein, we first reported the negative correlations between birthweight and total cholesterol and LDL-C levels in MODY2 patients. Thus, birthweight may be used as an early indicator to predict the disease progression of Chinese patients with MODY2.

Interestingly, when comparing clinical parameters according to the origins of the mutations, we found patients who inherited the mutations from their mothers had a significantly younger age of hyperglycemic onset and MODY2 diagnosis. On the one hand, maternal hyperglycemia during pregnancy may play an important role in the phenotypes of the baby. On the other hand, for MODY2 patients who inherit the mutations from their fathers, they may not be diagnosed with diabetes before screening, as they only have mild hyperglycemia, thus, this may lead to an older age of diagnosis, although the age of hyperglycemic onset may be earlier. The results suggest that glucose monitoring is necessary for babies with low birthweight.

Corresponding with birthweight results, the lipid profiles of the subjects with lower birthweight showed significantly higher total cholesterol and LDL-C levels when they inherited the mutations from their fathers and well-treated mothers during pregnancy than those from untreated mothers after adjusting for confounders including BMI. Although diabetes-related complications are rare in subjects with MODY2, dyslipidemia can indeed significantly increase the risk of cardiovascular diseases. Thus, our results indicated that lower birthweight might be a potential indicator for later metabolic conditions in patients with MODY2, and an improper treatment in pregnancy may result in harmful outcomes. Thus, fetal genotyping is recommended in pregnant MODY2 mothers.

To our knowledge, this is the first study to explicate the relationship between birthweight and metabolic traits in a Chinese MODY2 cohort. Nevertheless, there are several limitations that should be noted. First, Sanger sequencing may miss large deletions, thus, leading to an underestimate of the prevalence of MODY2, and multiplex-ligation dependent probe amplification may contribute to a more accurate diagnosis in the future. Second, although most of the patients were young and born in hospital, birthweight information was obtained from maternal recall rather than obstetric records, which may influence the accuracy of the data. Third, due to the low prevalence of MODY2, in order to increase the statistical power, patients with a wide age range were included in our study. Although we adjusted for age when performing analyses to minimize this limitation, further study with stricter inclusion criteria may be needed. Additionally, the patients included in our sample were relatively young and had a short duration of diabetes, which may be the reason for the negative correlation between birthweight and insulin resistance in our study, longer longitudinal studies are warranted for further evaluation. Finally, although we found associations of birthweight with metabolic indexes of MODY2 patients in China, it is not clear whether these results are further generalizable to the entire world. Therefore, prospective studies in different age strata of different races will be needed to clarify the relationship between birthweight and cardiometabolic risk in MODY2.

In summary, our study suggests that low birthweight in MODY2 patients might have harmful effects on later metabolic health. Therefore, MODY2 patients with low birthweight are advised to receive more active monitoring and intervention. Importantly, prenatal genetic diagnosis is strongly recommended in pregnant women diagnosed with MODY2.

## Supplementary information

Supplementary Table 1

Supplementary Table 2

Supplementary Table 3

Supplementary Table 4

## Abbreviations

MODY2: maturity-onset diabetes of the young type 2
HOMA-IR: homeostasis model assessment of insulin resistance
LDL-C: low density lipoprotein cholesterol
HDL-C: high-density lipoprotein cholesterol
