# Supplementary material for: Birthweight correlates with later metabolic abnormalities in Chinese patients with maturity-onset diabetes of the young type 2
Source: Endocrine. 2019 Apr 26;65(1):53–60. doi: 10.1007/s12020-019-01929-6 (PMC6606659; doi:10.1007/s12020-019-01929-6)
Supplement: Supplementary file 1 — Supplementary Table 1 [file 12020_2019_1929_MOESM1_ESM.docx]

| **Supplementary Table 1.** Primer sequence of *GCK* | | | | |
| --- | --- | --- | --- | --- |
| Exon | Forward | Reverse | Length | Temperature |
| 1 | TTGCCACCAGTCCCAGTT | ACTCCCAGAATGCCCAAT | 696 | 58.6/55.7 |
| 2 | GTGCAGATGCCTGGTGAC | AGGAGCCAAGGGTGAGAA | 411 | 57.8/57.4 |
| 3 | CTCCCTTAGTCCCTTGTGC | TCCCCACCCCTGGTAGACA | 385 | 56.6/61.7 |
| 4 | AAGCAGCAGCGGAAGAGG | GGCTACATTTGAAGGCAGAGT | 360 | 59.6/57.4 |
| 5/6 | TGCAGGAGGTAGTGACAGG | ACAGGGAGCCTCAGCAGT | 432 | 57.9/60.2 |
| 7 | AGAGGGACTCCTGTGGGC | CGGATTGTCAGTTTGCTTTT | 397 | 60.3/54.0 |
| 8 | CGAGGGAAAGACGTGAACC | CGTCGCCCTGAGACCAAG | 396 | 57.3/59.4 |
| 9 | GGCTCAGCGAGGGAAAGAG | TTGGGAACCGCAAGGAAC | 449 | 59.5/57.4 |
| 10 | TTTCGTAGTCCTCTTCTCGTCC | ATGGAGCCTGGGTGCTGT | 333 | 58.1/61.0 |
