# Supplementary material for: Birthweight correlates with later metabolic abnormalities in Chinese patients with maturity-onset diabetes of the young type 2
Source: Endocrine. 2019 Apr 26;65(1):53–60. doi: 10.1007/s12020-019-01929-6 (PMC6606659; doi:10.1007/s12020-019-01929-6)
Supplement: Supplementary file 2 — Supplementary Table 2 [file 12020_2019_1929_MOESM2_ESM.docx]

| **Supplementary Table 2.** Mutations found in GCK genes in our study | | |
| --- | --- | --- |
| Gene | Location | Nucleotide change |
| Novel mutations found in our study | | |
| GCK | Exon 10 | c.1334G>C |
| GCK | Exon 10 | c.1289_1294delTGACGC |
| GCK | Exon 6 | c.584T>C |
| GCK | Exon 1 | c.30delC |
| Reported mutations found in our study | | |
| GCK | Exon 9 | c.1220G>T |
| GCK | Exon 9 | c.1183G>T |
| GCK | Exon 9 | c.1136C>A |
| GCK | Exon 7 | c.781G>C |
| GCK | Exon 7 | c.776C>T |
| GCK | Exon 7 | c.755G>A |
| GCK | Exon 7 | c.683C>T |
| GCK | Exon 7 | c.683C>T |
| GCK | Exon 6 | c.667G>A |
| GCK | Exon 6 | c.645C>A |
| GCK | Exon 6 | c.630G>A |
| GCK | Exon 5 | c.571C>T |
| GCK | Exon 5 | c.556C>T |
| GCK | Exon 5 | c.532_532delG |
| GCK | Exon 5 | c.507G>C |
| GCK | Exon 4 | c.502A>G |
| GCK | Exon 4 | c.452_454delCCT |
| GCK | Exon 4 | c.370G>C |
| GCK | Exon 2 | c.130G>A |
| GCK | Exon 2 | c.127C>T |
| GCK | Exon 2 | c.118G>A |
