# Supplementary material for: Birthweight correlates with later metabolic abnormalities in Chinese patients with maturity-onset diabetes of the young type 2
Source: Endocrine. 2019 Apr 26;65(1):53–60. doi: 10.1007/s12020-019-01929-6 (PMC6606659; doi:10.1007/s12020-019-01929-6)
Supplement: Supplementary file 3 — Supplementary Table 3 [file 12020_2019_1929_MOESM3_ESM.docx]

| **Supplementary Table 3.** Partial correlations between birthweight and metabolic traits. | | | | |
| --- | --- | --- | --- | --- |
| *Variables* | *Unadjusted* | | *Adjusted age, gender and BMI* | |
|  | *r* | *P* | *r* | *P* |
| Age of MODY2 diagnosis (years) | 0.061 | 0.644 | / | / |
| Body mass index (kg/m2) | 0.235 | 0.090 | / | / |
| Age of hyperglycemic onset(years) | 0.079 | 0.542 | 0.184 | 0.200 |
| Duration of diabetes (years) | -0.040 | 0.764 | -0.154 | 0.287 |
| Systolic blood pressure (mmHg) | 0.036 | 0.839 | 0.056 | 0.763 |
| Diastolic blood pressure (mmHg) | 0.046 | 0.793 | 0.002 | 0.993 |
| Fasting glucose (mmol/L) | -0.192 | 0.172 | -0.205 | 0.167 |
| 2h- postprandial glucose (mmol/L) | -0.361 | **0.014** | -0.426 | **0.006** |
| Fasting insulin (mU/L) ^#^ | 0.123 | 0.568 | -0.006 | 0.981 |
| 2h- postprandial insulin(mU/L) ^#^ | -0.257 | 0.236 | -0.409 | 0.092 |
| Fasting C-peptide (ng/ml) | 0.154 | 0.401 | -0.109 | 0.588 |
| 2h- postprandial C-peptide (ng/ml) | 0.238 | 0.205 | 0.115 | 0.602 |
| Glycated albumin% | -0.441 | **0.019** | -0.462 | **0.035** |
| Glycated hemoglobin% | -0.44 | **0.003** | -0.529 | **0.001** |
| HOMA-IR | 0.146 | 0.496 | 0.023 | 0.925 |
| Total cholesterol (mmol/L) | -0.341 | **0.039** | -0.430 | **0.016** |
| Triglyceride (mmol/L) | -0.089 | 0.600 | -0.178 | 0.339 |
| HDL-C (mmol/L) | -0.076 | 0.656 | -0.047 | 0.803 |
| LDL-C (mmol/L) | -0.288 | 0.084 | -0.383 | **0.033** |
| Hs-CRP (mg/L) | 0.137 | 0.439 | 0.002 | 0.993 |
| Alanine transaminase (U/L) ^#^ | 0.110 | 0.504 | -0.049 | 0.789 |
| Aspartate aminotransferase (U/L) ^#^ | 0.080 | 0.761 | 0.178 | 0.560 |
| Creatinine (umol/L) ^#^ | 0.101 | 0.553 | 0.026 | 0.891 |
| Uric acid (umol/L) ^#^ | 0.049 | 0.786 | -0.012 | 0.952 |

Notes: r: partial correlation coefficients.

^#^ Skewed distributions were logarithmicallytransformed.

Values in bold are significant at *P*< 0.05.

Abbreviations: BMI, body mass index; MODY2, maturity onset diabetes of the young type 2; HOMA-IR, homeostasis model assessment of insulin resistance; LDL-C, low densitylipoprotein cholesterol; HDL-C, high-density lipoprotein cholesterol; Hs-CRP, high sensitive C-reactive protein.
