# Supplementary material for: Birthweight correlates with later metabolic abnormalities in Chinese patients with maturity-onset diabetes of the young type 2
Source: Endocrine. 2019 Apr 26;65(1):53–60. doi: 10.1007/s12020-019-01929-6 (PMC6606659; doi:10.1007/s12020-019-01929-6)
Supplement: Supplementary file 4 — Supplementary Table 4 [file 12020_2019_1929_MOESM4_ESM.docx]

| **Supplementary Table 4.** Clinical characteristics of MODY2 cases classified by origin of the mutation (n = 46) | | | | |
| --- | --- | --- | --- | --- |
| Laboratory (serum) | Father (n=18) | Mother (n= 28) | *P* |  |
| N (male/female) | 6/12 | 13/15 |  |  |
| Age of hyperglycemiconset (years) | 24.78 ± 10.09 | 15.96 ± 14.72 | **0.027** |  |
| Ageof MODY2 diagnosis (years) | 28.81 ± 13.58 | 18.21 ± 15.33 | **0.031** |  |
| Duration of diabetes (years) | 4.56 ± 4.94 | 2.25 ± 3.15 | 0.106 |  |
| Birthweight (kg) | 3.07 ± 0.32 | 3.24 ± 0.45 | 0.151 |  |
| Body mass index (kg/m2) | 19.83 ± 4.17 | 18.76 ± 4.35 | 0.463 |  |
| Systolic blood pressure (mmHg) | 103.46 ± 13.29 | 106.45 ± 17.50 | 0.647 |  |
| Diastolic blood pressure (mmHg) | 69.08 ± 12.91 | 66.00 ± 9.17 | 0.504 |  |
| Fasting glucose (mmol/L) | 6.81 ± 0.64 | 6.65 ± 0.55 | 0.419 |  |
| 2h- postprandial glucose (mmol/L) | 9.48 ± 1.97 | 8.57 ± 1.22 | 0.097 |  |
| Fasting insulin (mU/L) ^#^ | 1.73 ± 0.96 | 1.55 ± 0.45 | 0.613 |  |
| 2h- postprandial insulin (mU/L) ^#^ | 3.12 ± 0.84 | 3.29 ± 0.67 | 0.637 |  |
| Fasting C-peptide (ng/ml) | 0.98 ± 0.43 | 0.83 ± 0.42 | 0.369 |  |
| 2h- postprandial C-peptide (ng/ml) | 4.21 ± 2.02 | 3.25 ± 2.18 | 0.277 |  |
| Glycated albumin (%) | 17.60 ± 1.58 | 16.85 ± 2.54 | 0.394 |  |
| Glycated hemoglobin (%) | 6.45 ± 0.50 | 6.25 ± 0.35 | 0.165 |  |
| HOMA-IR | 0.50 ± 0.26 | 0.46 ± 0.15 | 0.719 |  |
| Total cholesterol (mmol/L) | 3.94 ± 0.64 | 4.33 ± 0.85 | 0.178 |  |
| Triglyceride (mmol/L) | 0.84 ± 0.47 | 0.55 ± 0.22 | **0.029** |  |
| HDL-C (mmol/L) | 1.40 ± 0.27 | 1.46 ± 0.26 | 0.518 |  |
| LDL-C (mmol/L) | 2.05 ± 0.73 | 2.49 ± 0.71 | 0.110 |  |
| Hs-CRP (mg/L) | 0.43 ± 0.50 | 0.30 ± 0.29 | 0.382 |  |

^#^ Skewed distributions were logarithmically transformed.

Abbreviations: MODY2, maturity onset diabetes of the young type 2; HOMA-IR, homeostasis model assessment of insulin resistance; LDL-C, low density lipoprotein cholesterol; HDL-C, high-density lipoprotein cholesterol; Hs-CRP, high sensitive C-reactive protein.
